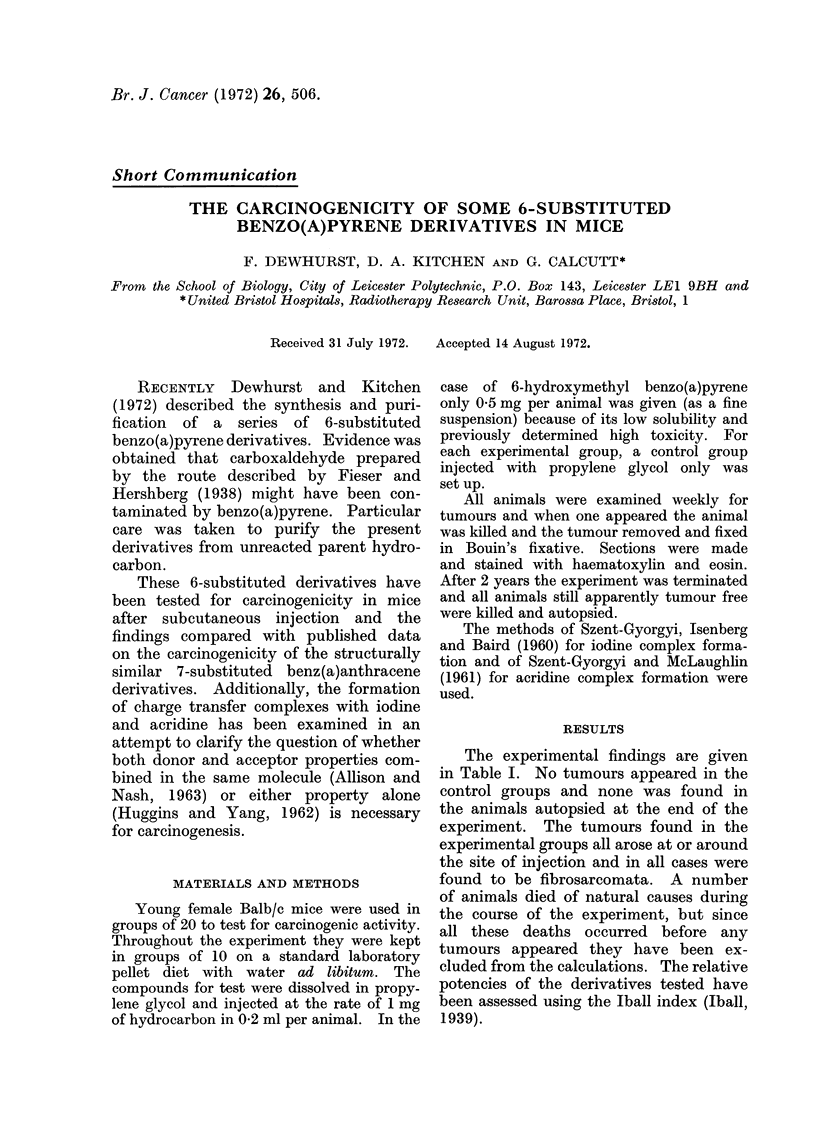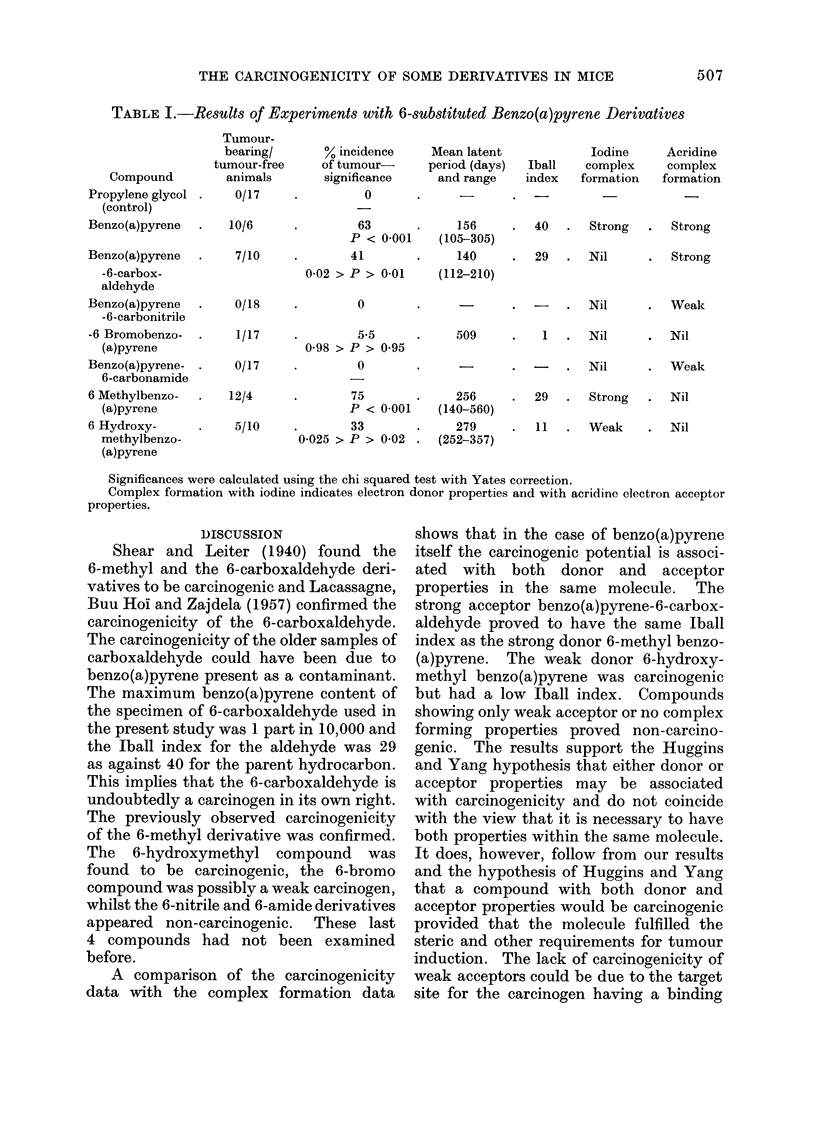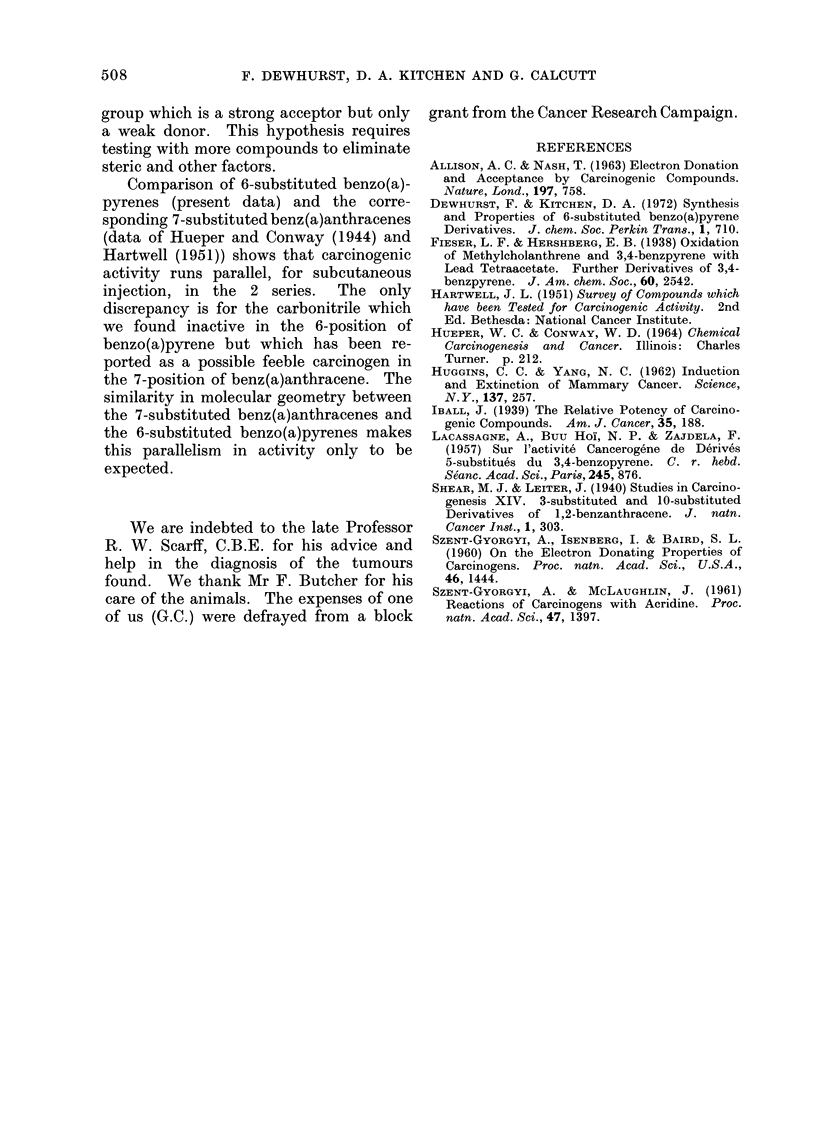# The carcinogenicity of some 6-substituted benzo(a)pyrene derivatives in mice.

**DOI:** 10.1038/bjc.1972.69

**Published:** 1972-12

**Authors:** F. Dewhurst, D. A. Kitchen, G. Calcutt


Br. J. Cancer (1972) 26, 506.

*Short Communication*

### THE CARCINOGENICITY OF SOME 6-SUBSTITUTED BENZO(A)PYRENE DERIVATIVES IN MICE

F. DEWHURST, D. A. KITCHEN AND G. CALCUTT*

*From the School of Biology, City of Leicester Polytechnic, P.O. Box 143, Leicester LE1 9BH and*
**United Bristol Hospitals, Radiotherapy Research Unit, Barossa Place, Bristol, 1*

Received 31 July 1972. Accepted 14 August 1972.

RECENTLY Dewhurst and Kitchen (1972) described the synthesis and purification of a series of 6-substituted benzo(a)pyrene derivatives. Evidence was obtained that carboxaldehyde prepared by the route described by Fieser and Hershberg (1938) might have been contaminated by benzo(a)pyrene. Particular care was taken to purify the present derivatives from unreacted parent hydrocarbon.

These 6-substituted derivatives have been tested for carcinogenicity in mice after subcutaneous injection and the findings compared with published data on the carcinogenicity of the structurally similar 7-substituted benz(a)anthracene derivatives. Additionally, the formation of charge transfer complexes with iodine and acridine has been examined in an attempt to clarify the question of whether both donor and acceptor properties combined in the same molecule (Allison and Nash, 1963) or either property alone (Huggins and Yang, 1962) is necessary for carcinogenesis.

### MATERIALS AND METHODS

Young female Balb/c mice were used in groups of 20 to test for carcinogenic activity. Throughout the experiment they were kept in groups of 10 on a standard laboratory pellet diet with water *ad libitum*. The compounds for test were dissolved in propylene glycol and injected at the rate of 1 mg of hydrocarbon in 0·2 ml per animal. In the case of 6-hydroxymethyl benzo(a)pyrene only 0·5 mg per animal was given (as a fine suspension) because of its low solubility and previously determined high toxicity. For each experimental group, a control group injected with propylene glycol only was set up.

All animals were examined weekly for tumours and when one appeared the animal was killed and the tumour removed and fixed in Bouin's fixative. Sections were made and stained with haematoxylin and eosin. After 2 years the experiment was terminated and all animals still apparently tumour free were killed and autopsied.

The methods of Szent-Gyorgyi, Isenberg and Baird (1960) for iodine complex formation and of Szent-Gyorgyi and McLaughlin (1961) for acridine complex formation were used.

### RESULTS

The experimental findings are given in Table I. No tumours appeared in the control groups and none was found in the animals autopsied at the end of the experiment. The tumours found in the experimental groups all arose at or around the site of injection and in all cases were found to be fibrosarcomata. A number of animals died of natural causes during the course of the experiment, but since all these deaths occurred before any tumours appeared they have been excluded from the calculations. The relative potencies of the derivatives tested have been assessed using the Iball index (Iball, 1939).

TABLE I.—*Results of Experiments with 6-substituted Benzo(a)pyrene Derivatives*

| Compound | Tumour-bearing/ tumour-free animals | % incidence of tumour—significance | Mean latent period (days) and range | Iball index | Iodine complex formation | Acridine complex formation |
|---|---|---|---|---|---|---|
| Propylene glycol (control) | 0/17 | 0<br>— | — | — | — | — |
| Benzo(a)pyrene | 10/6 | 63<br>$P < 0{\cdot}001$ | 156<br>(105–305) | 40 | Strong | Strong |
| Benzo(a)pyrene -6-carbox-aldehyde | 7/10 | 41<br>$0{\cdot}02 > P > 0{\cdot}01$ | 140<br>(112–210) | 29 | Nil | Strong |
| Benzo(a)pyrene -6-carbonitrile | 0/18 | 0 | — | — | Nil | Weak |
| -6 Bromobenzo-(a)pyrene | 1/17 | 5·5<br>$0{\cdot}98 > P > 0{\cdot}95$ | 509 | 1 | Nil | Nil |
| Benzo(a)pyrene-6-carbonamide | 0/17 | 0<br>— | — | — | Nil | Weak |
| 6 Methylbenzo-(a)pyrene | 12/4 | 75<br>$P < 0{\cdot}001$ | 256<br>(140–560) | 29 | Strong | Nil |
| 6 Hydroxy-methylbenzo-(a)pyrene | 5/10 | 33<br>$0{\cdot}025 > P > 0{\cdot}02$ | 279<br>(252–357) | 11 | Weak | Nil |

Significances were calculated using the chi squared test with Yates correction.
Complex formation with iodine indicates electron donor properties and with acridine electron acceptor properties.

### DISCUSSION

Shear and Leiter (1940) found the 6-methyl and the 6-carboxaldehyde derivatives to be carcinogenic and Lacassagne, Buu Hoï and Zajdela (1957) confirmed the carcinogenicity of the 6-carboxaldehyde. The carcinogenicity of the older samples of carboxaldehyde could have been due to benzo(a)pyrene present as a contaminant. The maximum benzo(a)pyrene content of the specimen of 6-carboxaldehyde used in the present study was 1 part in 10,000 and the Iball index for the aldehyde was 29 as against 40 for the parent hydrocarbon. This implies that the 6-carboxaldehyde is undoubtedly a carcinogen in its own right. The previously observed carcinogenicity of the 6-methyl derivative was confirmed. The 6-hydroxymethyl compound was found to be carcinogenic, the 6-bromo compound was possibly a weak carcinogen, whilst the 6-nitrile and 6-amide derivatives appeared non-carcinogenic. These last 4 compounds had not been examined before.

A comparison of the carcinogenicity data with the complex formation data shows that in the case of benzo(a)pyrene itself the carcinogenic potential is associated with both donor and acceptor properties in the same molecule. The strong acceptor benzo(a)pyrene-6-carboxaldehyde proved to have the same Iball index as the strong donor 6-methyl benzo-(a)pyrene. The weak donor 6-hydroxymethyl benzo(a)pyrene was carcinogenic but had a low Iball index. Compounds showing only weak acceptor or no complex forming properties proved non-carcinogenic. The results support the Huggins and Yang hypothesis that either donor or acceptor properties may be associated with carcinogenicity and do not coincide with the view that it is necessary to have both properties within the same molecule. It does, however, follow from our results and the hypothesis of Huggins and Yang that a compound with both donor and acceptor properties would be carcinogenic provided that the molecule fulfilled the steric and other requirements for tumour induction. The lack of carcinogenicity of weak acceptors could be due to the target site for the carcinogen having a binding group which is a strong acceptor but only a weak donor. This hypothesis requires testing with more compounds to eliminate steric and other factors.

Comparison of 6-substituted benzo(a)-pyrenes (present data) and the corresponding 7-substituted benz(a)anthracenes (data of Hueper and Conway (1944) and Hartwell (1951)) shows that carcinogenic activity runs parallel, for subcutaneous injection, in the 2 series. The only discrepancy is for the carbonitrile which we found inactive in the 6-position of benzo(a)pyrene but which has been reported as a possible feeble carcinogen in the 7-position of benz(a)anthracene. The similarity in molecular geometry between the 7-substituted benz(a)anthracenes and the 6-substituted benzo(a)pyrenes makes this parallelism in activity only to be expected.

We are indebted to the late Professor R. W. Scarff, C.B.E. for his advice and help in the diagnosis of the tumours found. We thank Mr F. Butcher for his care of the animals. The expenses of one of us (G.C.) were defrayed from a block grant from the Cancer Research Campaign.

### REFERENCES

ALLISON, A. C. & NASH, T. (1963) Electron Donation and Acceptance by Carcinogenic Compounds. *Nature, Lond.*, **197**, 758.

DEWHURST, F. & KITCHEN, D. A. (1972) Synthesis and Properties of 6-substituted benzo(a)pyrene Derivatives. *J. chem. Soc. Perkin Trans.*, **1**, 710.

FIESER, L. F. & HERSHBERG, E. B. (1938) Oxidation of Methylcholanthrene and 3,4-benzpyrene with Lead Tetraacetate. Further Derivatives of 3,4-benzpyrene. *J. Am. chem. Soc.*, **60**, 2542.

HARTWELL, J. L. (1951) *Survey of Compounds which have been Tested for Carcinogenic Activity.* 2nd Ed. Bethesda: National Cancer Institute.

HUEPER, W. C. & CONWAY, W. D. (1964) *Chemical Carcinogenesis and Cancer.* Illinois: Charles Turner. p. 212.

HUGGINS, C. C. & YANG, N. C. (1962) Induction and Extinction of Mammary Cancer. *Science, N.Y.*, **137**, 257.

IBALL, J. (1939) The Relative Potency of Carcinogenic Compounds. *Am. J. Cancer*, **35**, 188.

LACASSAGNE, A., BUU HOÏ, N. P. & ZAJDELA, F. (1957) Sur l'activité Cancerogéne de Dérivés 5-substitués du 3,4-benzopyrene. *C. r. hebd. Séanc. Acad. Sci., Paris*, **245**, 876.

SHEAR, M. J. & LEITER, J. (1940) Studies in Carcinogenesis XIV. 3-substituted and 10-substituted Derivatives of 1,2-benzanthracene. *J. natn. Cancer Inst.*, **1**, 303.

SZENT-GYORGYI, A., ISENBERG, I. & BAIRD, S. L. (1960) On the Electron Donating Properties of Carcinogens. *Proc. natn. Acad. Sci., U.S.A.*, **46**, 1444.

SZENT-GYORGYI, A. & MCLAUGHLIN, J. (1961) Reactions of Carcinogens with Acridine. *Proc. natn. Acad. Sci.*, **47**, 1397.